# Health Conditions and Psychotic Experiences: Cross-Sectional Findings From the American Life Panel

**DOI:** 10.3389/fpsyt.2020.612084

**Published:** 2021-01-13

**Authors:** Hans Oh, Lee Smith, Ai Koyanagi

**Affiliations:** ^1^Suzanne Dworak Peck School of Social Work, University of Southern California, Los Angeles, CA, United States; ^2^The Cambridge Centre for Sport and Exercise Sciences, Anglia Ruskin University, Cambridge, United Kingdom; ^3^Research and Development Unit, Centro de Investigación Biomédica en Red de Salud Mental (CIBERSAM), Parc Sanitari Sant Joan de Déu, Barcelona, Spain; ^4^Catalan Institution for Research and Advanced Studies (ICREA), Barcelona, Spain

**Keywords:** psychotic experiences, chronic health condition, multimorbidity, psychosis, prevention

## Abstract

**Background:** People with psychotic disorders have poor health, but studies have shown that people who have a milder and more prevalent form of psychosis (psychotic experiences) are also at risk for health problems. More research is needed to examine a broad range of health conditions to discover new relations with psychotic experiences.

**Methods:** We analyzed cross-sectional data from the American Life Panel, a nationally representative sample of the United States adult population. Using multivariable logistic regression, we examined the associations between health conditions (categories of conditions, specific conditions, count of conditions) and lifetime psychotic experiences.

**Results:** Approximately 71% of the weighted sample reported at least one health condition, and around 18% reported a lifetime psychotic experience. Using multivariable logistic regression, we found that several health conditions were associated with psychotic experiences, including pain due to other causes, neck pain, other injury, any gastrointestinal/kidney problem, liver diseases/cirrhosis, any nervous/sensory problem, migraine, nerve problem causing numbness/pain, any other disorder, specifically sleep disorder, chronic fatigue syndrome, and chronic pain. Further, the count of specific health conditions and the count of categories were associated with greater odds of psychotic experiences.

**Conclusion:** We found that numerous health conditions were associated with psychotic experiences.

## Introduction

Hallucinatory experiences and delusional ideations can occur in the general population without being distressful or impairing to a clinically significant degree (1). The prevalence of psychotic experiences has been estimated to be around 7%, though it can be higher or lower depending on the country (2–4). Most people who report psychotic experiences only experience them on a few occasions: One large study consisting of 31,261 adults from 18 countries found that 32.2% of respondents with lifetime psychotic experiences reported only one occurrence, and 31.8% reported only two to five occurrences (3). Moreover, most people who have psychotic experiences do not develop a psychotic disorder [though prior studies have shown psychotic experiences are associated with more persistent forms of psychosis (5)]. Still, psychotic experiences have been a topic of public health discussion because they are cross-sectionally associated with numerous negative mental and physical health outcomes, including disability (6, 7), poor quality of life (8), suicidal thoughts and behaviors (9), medication consumption and hospital admissions (10), and premature mortality (11).

While it is widely accepted that people with psychotic disorders are at risk for chronic health conditions (12) and shorter lives (13, 14), an emerging body of literature has shown that those with psychotic experiences are also at risk for poor physical health. Moreno et al. (10) analyzed the World Health Organization (WHO) World Health Survey, and found that angina, asthma, arthritis, tuberculosis, vision or hearing problems, mouth/teeth problems, alcohol consumption, smoking, and accidents were associated with psychotic experiences, with the number of health conditions increasing with the number of psychotic experiences. Using these same data, Stubbs et al. (12) found that in 48 low- and middle-income countries, psychotic experiences were associated with 2.20 times greater odds for multimorbidity (aOR: 2.20; 95% CI: 2.02–2.39). Scott et al. (15) analyzed the WHO World Mental Health Surveys and found that psychotic experiences were significantly associated with subsequent onset of arthritis, back or neck pain, frequent or severe headache, other chronic pain, heart disease, high blood pressure, diabetes and peptic ulcer. In the United States, Oh et al. (16, 17) found that certain health conditions (stroke, epilepsy, arthritis, frequent/severe headaches, chronic back/neck pain, other chronic pain, heart disease, high blood pressure, asthma, ulcers, fertility problems, hearing problems, vision problems, allergies/infections) were related to psychotic experiences, with varying levels of strength and significance depending on the racial/ethnic group, and with evidence that the odds of psychotic experiences increased with the number of health conditions in a dose-response fashion.

### Aims of the Study

In this study, we built on existing literature by analyzing data from a representative sample of the United States general population to study the associations between physical health conditions and psychotic experiences, using a wide range of conditions, many of which have yet to be examined in relation to psychotic experiences.

## Methods

### Sample

This study analyzed publicly available data from the RAND American Life Panel (ALP) (18), which is a US nationally representative probability-based panel consisting of ~6,000 adults aged 18 and older. These panel members were recruited using probability sampling methods [e.g., address-based samples and telephone (random-digit dial) samples]. To enhance representativeness, RAND provides computers and internet service to members who would not otherwise be able to participate. In 2019, RAND administered two surveys drawing respondents from the panel: the first was the ALP Omnibus Survey (*N* = 2,555) conducted in February-April, which had a completion rate of 64.9%; and the second was the Health and Functional Capacity Survey (*N* = 2,657) conducted April–June, which had a completion rate of 78.2%. All of the panel members who completed the ALP Omnibus Survey were included in the Health and Functional Capacity Survey, allowing us to merge the two data sets to yield a final sample of *N* = 2,554 for the current study. Sampling weights were constructed to account for non-response and probability of selection using population distributions from the Current Population Survey Annual Social and Economic Supplement (provided by the U.S. Census Bureau). Data collection and survey protocols were approved by RAND's Human Subjects Protection Committee, which serves as RAND's Institutional Review Board.

### Measures

#### Lifetime Psychotic Experiences (Dependent)

Psychotic experiences were measured using an abbreviated version of the WHO Composite International Diagnostic Interview (CIDI) Psychosis Screen, which has been used in large global epidemiology studies (3). Respondents were asked if they had ever experienced the following: (1) A feeling something strange and unexplainable was going on that other people would find hard to believe?; (2) A feeling that people were too interested in you or that there was a plot to harm you?; (3) A feeling that your thoughts were being directly interfered or controlled by another person, or your mind was being taken over by strange forces?; and (4) An experience of seeing visions or hearing voices that others could not see or hear when you were not half asleep, dreaming, or under the influence of alcohol or drugs? Endorsing any of these experiences constituted lifetime psychotic experience.

#### Health Conditions (Independent)

Respondents were asked (yes/no): “Do you suffer from any of the following serious health problems?” The list of conditions included 10 categories of disorders, which were: (1) Blood disorders; (2) Bone, joint, muscle, skin problems; (3) Cancer; (4) Diabetes; (5) Gastrointestinal or kidney problems; (6) Heart of circulatory problems; (7) Infectious diseases; (8) Lung problems; (9) Nervous or sensory problems; and (10) other disorders. Each category contained specific conditions, which are listed in Table 1.

**Table 1:** Prevalence of physical health conditions among people with and without lifetime psychotic experiences.

	**Total**	**PE**	**No PE**	**Chi-square**	***P*-value**
**Condition**	***N* (%)**	***N* (%)**	***N* (%)**		
**Blood disorders**					
Any blood disorder	52 (2.04)	13 (2.90)	39 (1.85)	2.04	0.15
Sickle cell anemia	5 (<0.00)	4 (0.95)	1 (0.21)	13.68	0.00
Other blood disorder	47 (1.93)	9 (2.13)	38 (1.89)	0.10	0.75
**Bone, joint, muscle or skin problem**					
Any bone, joint, muscle or skin problem	1337 (52.35)	264 (58.93)	1073 (50.95)	9.43	0.00
Arthritis	782 (32.11)	145 (34.28)	637 (31.68)	1.09	0.27
Rheumatoid arthritis	81 (3.33)	16 (3.78)	65 (3.23)	0.33	0.55
Back pain due to spinal stenosis	167 (6.86)	30 (7.09)	137 (6.81)	0.04	0.83
Back pain due to other causes	663 (27.24)	166 (39.24)	497 (24.71)	37.22	0.00
Neck pain	399 (16.39)	117 (27.66)	282 (14.02)	47.42	0.00
Fibromyalgia	83 (3.41)	24 (5.67)	59 (2.93)	7.96	0.00
Lupus	20 (0.82)	5 (1.18)	15 (0.75)	0.81	0.39
Amputation of limb	13 (0.53)	3 (0.71)	10 (0.50)	0.30	0.52
Deformity of limb	8 (0.33)	1 (0.24)	7 (0.35)	0.13	0.72
Ehlers-Danlos syndrome	2 (<0.00)	1 (0.24)	1 (<0.00)	1.48	0.22
Severe burn	4 (0.16)	3 (0.71)	1 (<0.00)	9.26	0.00
Other bone or joint disorder	178 (7.31)	38 (8.98)	140 (6.96)	2.11	0.13
Other injury	139 (5.71)	37 (8.75)	102 (5.07)	8.76	0.00
Other muscle or connective tissue disorder	93 (3.82)	23 (5.44)	70 (3.48)	3.64	0.07
**Cancer**					
Cancer	101 (4.15)	15 (3.55)	86 (4.28)	0.47	0.51
**Diabetes**					
Diabetes	333 (13.68)	61 (14.42)	272 (13.53)	0.24	0.61
**Gastrointestinal or kidney problem**					
Any gastrointestinal or kidney problem	385 (15.07)	93 (20.76)	292 (13.87)	13.71	0.00
Chron's disease	15 (0.62)	6 (1.42)	9 (0.45)	5.38	0.01
Ulcerative colitis	24 (0.99)	8 (1.89)	16 (0.80)	4.30	0.03
Liver disease/cirrhosis	14 (0.58)	7 (1.65)	7 (0.35)	10.43	0.00
Chronic kidney disease (not on dialysis)	48 (1.97)	13 (3.07)	35 (1.74)	3.21	0.10
Chronic kidney disease (on dialysis)	7 (0.29)	2 (0.47)	5 (0.25)	0.61	0.43
Other gastrointestinal disorder	245 (10.07)	55 (13.00)	190 (9.45)	4.88	0.03
Other kidney or bladder disorder	75 (3.08)	17 (4.02)	58 (2.88)	1.50	0.24
**Heart or circulatory problem**					
Any heart or circulatory problem	320 (12.53)	56 (1.25)	264 (12.54)	0.00	0.98
Heart failure	48 (1.97)	8 (1.89)	40 (1.99)	0.02	0.89
Coronary artery disease	81 (3.33)	7 (1.65)	74 (3.68)	4.45	0.04
Heart valve dysfunction	35 (1.44)	7 (1.65)	28 (1.39)	0.17	0.71
Peripheral arterial disease	42 (1.73)	7 (1.65)	35 (1.74)	0.01	0.90
Abnormal heart rhythm	34 (1.40)	4 (0.95)	30 (1.49)	0.76	0.39
Lymphedema	24 (0.99)	6 (1.42)	18 (0.90)	0.98	0.34
Other heart or circulatory system disorder	152 (6.24)	34 (8.04)	118 (5.87)	2.81	0.10
**Infectious disease**					
Any infectious disease	51 (2.00)	15 (3.35)	36 (1.71)	5.07	0.02
HIV	19 (0.78)	7 (1.65)	12 (0.60)	5.05	0.02
Hepatitis	13 (0.53)	4 (0.95)	9 (0.45)	1.63	0.20
Other infectious diseases	21 (0.86)	5 (1.18)	16 (0.80)	0.61	0.43
**Lung problem**					
Any lung problem	341 (13.35)	83 (18.53)	258 (12.25)	12.58	0.00
Asthma	241 (9.90)	58 (13.71)	183 (9.10)	8.33	0.01
Chronic obstructive pulmonary disease/emphysema	76 (3.12)	13 (3.07)	63 (3.13)	0.00	0.95
Interstitial lung disease/pulmonary fibrosis	2 (<0.00)	0 (0.00)	2 (<0.00)	0.42	0.52
Pulmonary hypertension	33 (1.36)	8 (1.89)	25 (1.24)	1.10	0.30
Other lung disease	33 (1.36)	10 (2.36)	23 (1.14)	3.89	0.05
**Nervous or sensory problem**					
Any nervous or sensory problem	656 (25.69)	163 (36.38)	493 (23.41)	32.58	0.00
Spinal cord injury	24 (0.99)	6 (1.42)	18 (0.9)	0.98	0.39
Multiple sclerosis	18 (0.74)	5 (1.18)	13 (0.65)	1.36	0.26
Seizure disorder	10 (0.41)	1 (0.24)	9 (0.45)	0.38	0.54
Parkinson's disease	7 (0.29)	2 (0.47)	5 (0.25)	0.61	0.44
Stroke (or effects of a prior stroke)	36 (1.48)	9 (2.13)	27 (1.34)	1.48	0.21
Migraine	245 (10.07)	73 (17.26)	172 (8.55)	29.25	0.00
Blindness	13 (0.53)	1 (0.24)	12 (0.60)	0.85	0.36
Deafness	49 (2.01)	8 (1.89)	41 (2.04)	0.04	0.84
Nerve problem causing numbness or pain	349 (14.34)	91 (21.51)	258 (12.83)	21.45	0.00
Other nervous system disorder	58 (2.38)	17 (4.02)	41 (2.04)	5.90	0.02
**Other disorders**					
Any other disorder (obesity, sleep, pain, immune, other health)	804 (31.48)	195 (43.53)	609 (28.92)	36.55	0.00
Obesity	431 (17.71)	100 (23.64)	331 (16.46)	12.37	0.00
Sleep disorder	281 (11.54)	82 (19.39)	199 (9.90)	30.82	0.00
Chronic fatigue syndrome	48 (1.97)	19 (4.49)	29 (1.44)	16.81	0.00
Chronic pain	189 (7.76)	61 (14.42)	128 (6.36)	31.67	0.00
Immune deficiency	24 (0.99)	6 (1.42)	18 (0.90)	0.98	0.32
Other health problem	169 (6.94)	33 (7.80)	136 (6.76)	0.58	0.42

#### Sociodemographic Characteristics (Covariates)

Sociodemographic characteristics included age (18–25, 26–44, 45–64, 65+), sex (male, female), education (less than high school, some high school but no diploma, high school graduate or equivalent, some college but no degree, professional school degree, Associate's degree, Bachelor's degree, Master's degree, Doctoral degree), income (<$25,000, $25,000–49,999, $50,0000–74,999, $75,000–99,999, $100,000–124,999, $125,000–199,999, $200,000, or more), and race/ethnicity (White, Black, Latinx, Other). All sociodemographic characteristics can be found in Supplementary Table 1.

#### Psychiatric and Substance Use Disorders (Covariates)

Psychiatric disorders are highly comorbid with psychotic experiences (19), including depression and anxiety (19, 20), which are also associated to several physical health conditions (21). In keeping with prior studies (15–17), we adjusted for the presence of psychiatric and substance use disorders. Psychiatric disorder was coded dichotomously to reflect the presence of at least one of the following self-reported conditions: schizophrenia, bipolar disorder, depression, anxiety, attention deficit/hyperactivity disorder, post-traumatic stress disorder, Alzheimer's disease, other dementia, other mental or cognitive disorder. Substance use disorder was also coded dichotomously to reflect the presence of at least one of the following conditions: alcohol dependence, opioid dependence, other substance use disorder). The descriptive summary of psychiatric disorders and substance use disorders is presented in Supplementary Table 3.

### Analysis

**Figure 1:**
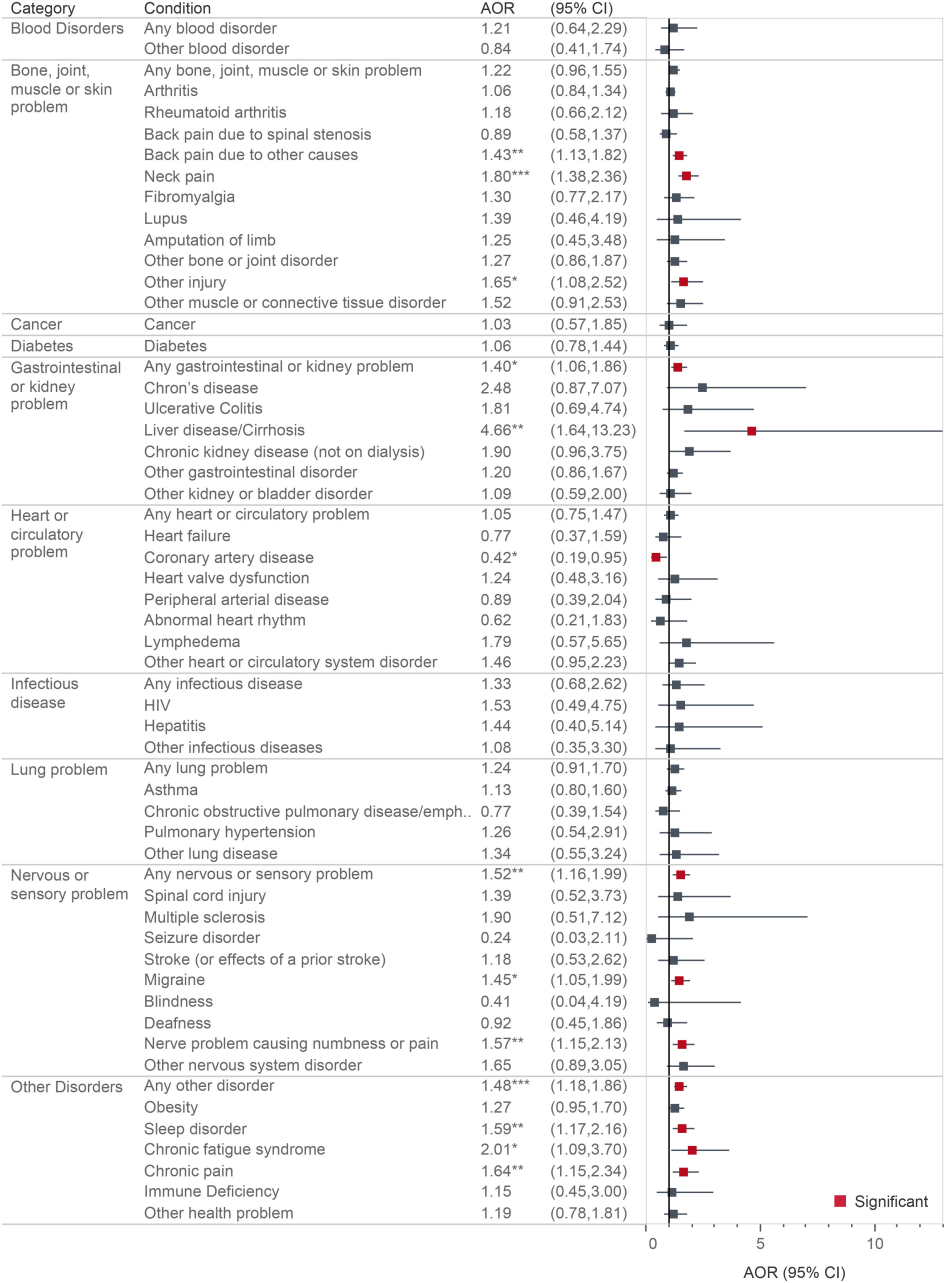
Multivariable logistic regression showing associations between health conditions and lifetime psychotic experiences. All models are adjusted for age, sex, education, income, race/ethnicity, psychiatric disorder, and substance use disorder. *p < 0.05; **p < 0.01; ***p < 0.001.

We calculated the total prevalence of health conditions and lifetime psychotic experiences. We then examined the prevalence of health conditions among those with and without psychotic experiences, testing whether the proportions were significantly different across categories. We then conducted simple bivariate logistic regression models examining the associations between each health condition and psychotic experiences (see Supplementary Table 2), and then conducted multivariable logistic regression models controlling for basic sociodemographic characteristics (age, sex, education, income, and race/ethnicity) (Supplementary Figure 1). Final models included full adjustments for sociodemographic characteristics, psychiatric disorder, and substance use disorder (Figure 1). We used complete case analyses, allowing sample sizes to vary according to data available, with <5% (*n* = 124) missing in any given model. Due to small cell counts (*n* < 10), several conditions could not produce reliable estimates, thus the following conditions were included in the broader categories but were not presented in Figure 1: severe burn, deformity of limb, Alzheimer's Disease, interstitial lung disease/pulmonary fibrosis, Ehlers-Danlos Syndrome, chronic kidney disease (on dialysis), and sickle cell anemia. We then examined the total count of specific health conditions and count of categories in relation to psychotic experiences.

## Results

### Descriptive Summary

About 70.68% (*N* = 1,806) of the weighted sample reported at least one health condition (95% CI; 68.59–72.70). The most common category of condition was bone/joint/muscle/skin problems, while the least common categories of conditions were infectious diseases and blood disorders. Approximately 17.54% (*N* = 448) reported a lifetime psychotic experience (95% CI 15.84–19.39). All sociodemographic characteristics of the sample can be found in Supplementary Table 1. In brief, around a fifth of the sample was young adults and over a third was older adults. The majority (~57%) of the sample was female and most of the sample was educated (~86% had at least some college or higher), with nearly 40% of the sample earning a household income of under $50,000 and over a third earning over $100,000. Most of the sample (~71%) was racially White. Over a quarter of the sample had at least one psychiatric or substance use disorder.

Bivariate associations between health conditions and lifetime psychotic experiences are shown in the Supplementary Table 2. Multivariable logistic regression models with simple adjustments for sociodemographic characteristics only (i.e., without adjustments for psychiatric disorder or substance use disorder) can also be found in the Supplementary Figure 1. All fully adjusted models are shown in Figure 1.

### Blood Disorders

Blood disorders were not significantly associated with psychotic experiences.

### Bone, Joint, Muscle, or Skin Problems

With only basic adjustments for sociodemographic characteristics, having any bone/joint/muscle/skin problem was associated with 1.52 times greater odds of psychotic experiences, adjusted for age, sex, education, income, and race (aOR: 1.52; 95% CI: 1.22–1.91). Specifically, these problems included arthritis, back pain (due to other causes), neck pain, fibromyalgia, other muscle/connective tissue disorder, and other injury. Certain conditions were not associated with psychotic experiences, including rheumatoid arthritis, back pain due to spinal stenosis, lupus, amputation of limb, and other bone/joint disorder. After adjustments for psychiatric disorder and substance use disorder, however, only back pain due to other causes (aOR: 1.43; 95% CI: 1.13–1.82), neck pain (aOR: 1.80; 95% CI: 1.38–2.36), and other injury (aOR: 1.65; 95% CI: 1.08–2.52) were significantly associated with psychotic experiences.

### Cancer

Cancer was not significantly associated with psychotic experiences.

### Diabetes

Diabetes was not significantly associated with psychotic experiences.

### Gastrointestinal or Kidney Problems

With only basic adjustments for sociodemographic characteristics, having any gastrointestinal/kidney problem was associated 1.59 times greater odds of psychotic experiences (aOR: 1.59; 95% CI: 1.21–2.08). Specific significant conditions were Crohn's Disease, liver disease/cirrhosis, and other gastrointestinal disorder, and non-significant conditions were ulcerative colitis, chronic kidney disease (on and off dialysis), or other kidney/bladder disorder. However, after adjustments for psychiatric disorders and substance use disorders, only any gastrointestinal or kidney problem (aOR 1.40; 95% CI: 1.06–1.86) and liver disease/cirrhosis (aOR: 4.66; 95% CI: 1.64–13.23) were significantly associated with psychotic experiences.

### Heart or Circulatory Problems

Having any heart/circulatory problem was not significantly associated with psychotic experiences, nor was any specific type of problem (e.g., failure, coronary artery disease, heart valve dysfunction, peripheral arterial disease, abnormal heart rhythm, and lymphedema), with the exception of “other heart/circulatory system disorder” (aOR: 1.66; 95% CI: 1.09–2.53). However, after adjustments for psychiatric disorder and substance use disorder, this association was no longer significant. Further, coronary artery disease was associated with lower odds of psychotic experiences (aOR: 0.42; 95% CI: 0.19–0.95).

### Infection Diseases

Having “any infectious disease” was not associated with psychotic experiences, except for Human Immunodeficiency Virus (HIV), which was no longer significant after adjustments for psychiatric disorder and substance use disorder.

### Lung Problems

With only adjustments of sociodemographic characteristics, having “any lung problem” was associated with 1.43 times greater odds of psychotic experiences (aOR: 1.43; 95% CI: 1.09–1.88), though specific significant conditions such as asthma, chronic obstructive pulmonary disease/emphysema, pulmonary hypertension, and other lung disease, were not associated with psychotic experiences. After adjustments for psychiatric disorder and substance use disorder, none of the lung problems were associated with psychotic experiences.

### Nervous or Sensory Problems

With only adjustments of sociodemographic characteristics, having any nervous/sensory problem was associated with 1.74 times greater odds of having psychotic experiences (aOR: 1.74; 95% CI: 1.34–2.25). Specific significant conditions included migraine, nerve problem causing numbness or pain, and other nervous system disorder. Conditions that were not associated with psychotic experiences were spinal cord injury, multiple sclerosis, seizure disorder, Parkinson's disease, stroke, blindness, and deafness. After adjustments for psychiatric disorders and substance use disorders, only any nervous or sensory problems (aOR: 1.52; 95% CI: 1.16–1.99), migraine (aOR: 1.45; 95% CI: 1.05–1.99), and nerve problem causing numbness or pain (aOR: 1.57; 95% CI: 1.15–2.13) were associated with psychotic experiences.

### Other Disorders

With only adjustments of sociodemographic characteristics, other health problems were associated with 1.88 times greater odds of psychotic experiences (aOR: 1.88; 95% CI: 1.30–2.36). Conditions in this category that were associated with psychotic experiences included obesity, sleep disorder, chronic fatigue syndrome, chronic pain, other health problem. Immune deficiency was not associated with psychotic experiences. After adjustments for psychiatric disorder and substance use disorder, only any other disorder (aOR: 1.48; 95% CI: 1.18–1.86), sleep disorder (aOR: 1.59; 95% CI: 1.17–2.16), chronic fatigue syndrome (aOR: 2.01; 95% CI: 1.09–3.70) and chronic pain (aOR: 1.64; 95% CI: 1.15–2.34) were significantly associated with psychotic experiences.

### Count of Conditions

Having any health condition was associated with 1.38 times greater odds of psychotic experiences (aOR: 1.38; 95% CI: 1.03–1.83), adjusting for sociodemographic characteristics, psychiatric disorder, and substance use disorder. A one-condition increase in the total number of specific conditions was associated with 1.09 times greater odds of psychotic experiences (aOR: 1.09; 95% CI: 1.05–1.13), and a one-category increase in the number of types of conditions was associated with a 1.18 times greater odds of psychotic experiences (aOR: 1.18; 95% CI: 1.09–1.28).

## Discussion

### Summary of Findings

In this representative sample of the U.S., we found that several physical health conditions were significantly associated with lifetime psychotic experiences, adjusting for sociodemographic characteristics. However, many associations attenuated and lost significance after adjusting for psychiatric disorder and substance use disorder. In the end, the only health conditions associated with psychotic experiences were specific bone/join/muscle/skin problems (such as back pain due to other causes, neck pain, other injury, but not any other specific condition within this category), any gastrointestinal/kidney problem (though not specific conditions within this category except for liver diseases/cirrhosis), any nervous/sensory problem (though not specific conditions within this category except for migraine and nerve problem causing numbness/pain), any other disorder (specifically sleep disorder, chronic fatigue syndrome, and chronic pain, but not other conditions within this category). Also, the continuous count of specific health conditions and categories of conditions were associated with greater odds of psychotic experiences. Our findings align with existing studies that show significant associations between psychotic experiences and a range of health conditions (15–17, 22). In particular, our study seemed to highlight prior findings that psychotic experiences are related to pain conditions (15, 16, 23–25). However, our study also offered novel contributions, as it was among the first (to our knowledge) to show that liver diseases/cirrhosis and chronic fatigue syndrome were related to psychotic experiences.

Numerous statistically significant associations were rendered non-significant after adjusting for sociodemographic characteristics, and even more so after adjusting for psychiatric disorder and substance use disorder, suggesting that much of the associations between health conditions and psychotic experiences may be partially explained by aspects of mental health. Ultimately, we did not find significant associations for several categories of health conditions, including blood disorders, cancer, diabetes, infectious diseases, and lung problems. It is possible that conditions were not significant due to a lack of statistical power, resulting in false negatives, which is notable since the majority of effect sizes generally suggested greater odds of psychotic experiences. The non-significant associations for cancer and diabetes is puzzling given that the health behaviors that contribute to these conditions are also related to psychotic experiences [e.g., tobacco use (26, 27)]. The non-significant association between cardiovascular diseases and psychotic experiences was also unexpected given prior studies (17, 28, 29). We also did not find that seizures or strokes were associated with psychotic experiences, which again, contradicted prior studies (16, 30). We were the first to use representative data to examine whether blindness was associated with psychotic experiences, and found null results likely due to the small cell count, though some have hypothesized that early blindness is actually protective against psychosis (31).

Health conditions are often inter-related and share risk factors, obfuscating the exact pathways by which specific physical health conditions and psychotic experiences are connected. Stress and chronic activation of the hypothalamic-pituitary-adrenal axis can have adverse effect on health (32–34) and may underlie the associations we found in our study. Further, given that we found several pain-related conditions were linked to psychotic experiences, inflammation may be implicated (35, 36). Along these lines, microglia (i.e., immune cells in the central nervous system that play a key role in inflammation) have been related to health problems (37, 38), and their activation can result in excessive synaptic pruning, loss of cortical gray matter, and loss of cortical control, which may lead to the disinhibition of subcortical dopamine and the occurrence of hallucinatory experiences (39, 40). However, the link between inflammation and psychotic experiences is not always clear (41) and future research should elucidate the physiological pathways by which health conditions and psychotic experiences are related.

### Limitations

This study has several limitations. First, the data are cross-sectional and did not allow us to ascertain the temporal order of events (i.e., whether psychotic experiences preceded the health conditions, or vice versa). Prospective cohort studies are needed to sort out the directionality of the associations we found in our study. Second, the CIDI was only used to assess psychotic experiences, and all other health conditions were self-reported and subject to biases. People may have had undiagnosed health problems or otherwise underreported their health conditions (especially if symptoms were mild). Future studies can employ clinical/structured interviews, or conduct surveys within healthcare systems so that administrative data can be used to link health conditions documented in medical records and insurance claims to psychotic experiences. Third, due to small cell counts, several associations may have been underpowered. For this reason, we were unable to examine some specific health conditions and were unable to differentiate the subtypes of psychotic experiences. It is possible that health outcomes may have been differentially related to hallucinatory experiences and delusional ideations (42).

### Conclusions and Implications

There is reason to believe that psychotic experiences are cross-sectional indicators of psychological distress (43, 44), as a meta-analysis found that people with psychotic experiences were more than twice as likely to report mental health service use when compared with people without psychotic experiences (45). Further, there is evidence to suggest that psychotic experiences predict subsequent contact with mental health services (46). Psychotic experiences may also be cross-sectional indicators of physical distress as well, as one study found that psychotic experiences were associated with several subsequent chronic health conditions (15). In agreement with prior cross-sectional studies, a significant portion of the general population will report having a psychotic experience at some point in life, and these experiences, as we have shown in this study, are linked to several physical health outcomes. Psychotic experiences are also associated with health behaviors (e.g., smoking, sleep disturbance), which are strongly predictive of subsequent health problems (47, 48). It is important to consider that psychotic experiences often happen for the first time in young adulthood [median age of 26 years old (49)], which is potentially years or even decades before symptoms of certain chronic health conditions emerge. However, there has been little translational work to assess the utility of psychotic experiences in predicting subsequent chronic health conditions. Thus, it has yet to be determined whether psychotic experiences can signal physical and psychological distress in such a way that they effectively guide the selection and timing of preventive interventions. In conclusion, more prospective cohort studies are needed to ascertain the predictive utility of psychotic experiences, and clinicians should be aware that many health conditions are associated with psychotic experiences.

## Data Availability Statement

The datasets generated for this study can be found in online repositories. The names of the repository/repositories and accession number(s) can be found at: https://www.rand.org/research/data/alp/data-access.html.

## Ethics Statement

The studies involving human participants were reviewed and approved by RAND Corporation. The patients/participants provided their written informed consent to participate in this study.

## Author Contributions

HO served as the lead writer for the manuscript. LS provided conceptual and editorial support. AK oversaw all statistical analyses and assisted with data interpretation. All authors contributed substantially to the development of this manuscript and have approved of this final submission.

## Conflict of Interest

The authors declare that the research was conducted in the absence of any commercial or financial relationships that could be construed as a potential conflict of interest.
