# Supplementary material for: Health Conditions and Psychotic Experiences: Cross-Sectional Findings From the American Life Panel
Source: Front Psychiatry. 2021 Jan 13;11:612084. doi: 10.3389/fpsyt.2020.612084 (PMC7839662; doi:10.3389/fpsyt.2020.612084)
Supplement: Supplementary file 3 [file Table_3.docx]

| **Table S3. Prevalence of psychiatric disorders and substance use disorders among people with and without lifetime psychotic experiences.** | | | | | |
| --- | --- | --- | --- | --- | --- |
|  | **Total** | **PE** | **No PE** | **Chi-square** | **P-value** |
| **Condition** | N (%) | N (%) | N (%) |  |  |
| **Psychiatric disorders and substance use disorders** |  |  |  |  |  |
| Any mental health or substance use problem | 666(26.08) | 216(48.21) | 450(21.37) | 138.12 | 0.00 |
| Schizophrenia | 9(0.37) | 8(1.89) | 1(<0.00) | 32.17 | 0.00 |
| Bipolar disorder | 56(2.30) | 31(7.33) | 25(1.24) | 57.58 | 0.00 |
| Depression | 432(17.75) | 157(37.12) | 275(13.67) | 131.55 | 0.00 |
| Anxiety | 442(18.16) | 152(35.93) | 290(14.42) | 108.83 | 0.00 |
| Attention Deficit/Hyperactivity Disorder | 71(2.92) | 27(6.38) | 44(2.19) | 21.72 | 0.00 |
| Post-traumatic stress disorder | 116(4.77) | 51(12.06) | 65(3.23) | 59.96 | 0.00 |
| Personality disorder | 5(0.21) | 4(0.95) | 1(<0.00) | 13.68 | 0.00 |
| Alzheimer’s disease | 1(<0.00) | 0(0.00) | 1(<0.00) | 0.21 | 0.64 |
| Other dementia | 13(0.53) | 7(1.65) | 6(0.30) | 12.10 | 0.00 |
| Other mental or cognitive disorder | 27(1.11) | 12(2.84) | 15(0.75) | 13.93 | 0.00 |
| Alcohol dependence | 32(1.31) | 9(2.13) | 23(1.14) | 2.61 | 0.09 |
| Opioid dependence | 13(0.53) | 4(0.95) | 9(0.45) | 1.63 | 0.20 |
| Other Substance Use Disorder | 13(0.53) | 6(1.42) | 7(0.35) | 7.54 | 0.01 |
|  |  |  |  |  |  |
