# Supplementary figures and images for: Health Conditions and Psychotic Experiences: Cross-Sectional Findings From the American Life Panel
Source: Front Psychiatry. 2021 Jan 13;11:612084. doi: 10.3389/fpsyt.2020.612084 (PMC7839662; doi:10.3389/fpsyt.2020.612084)

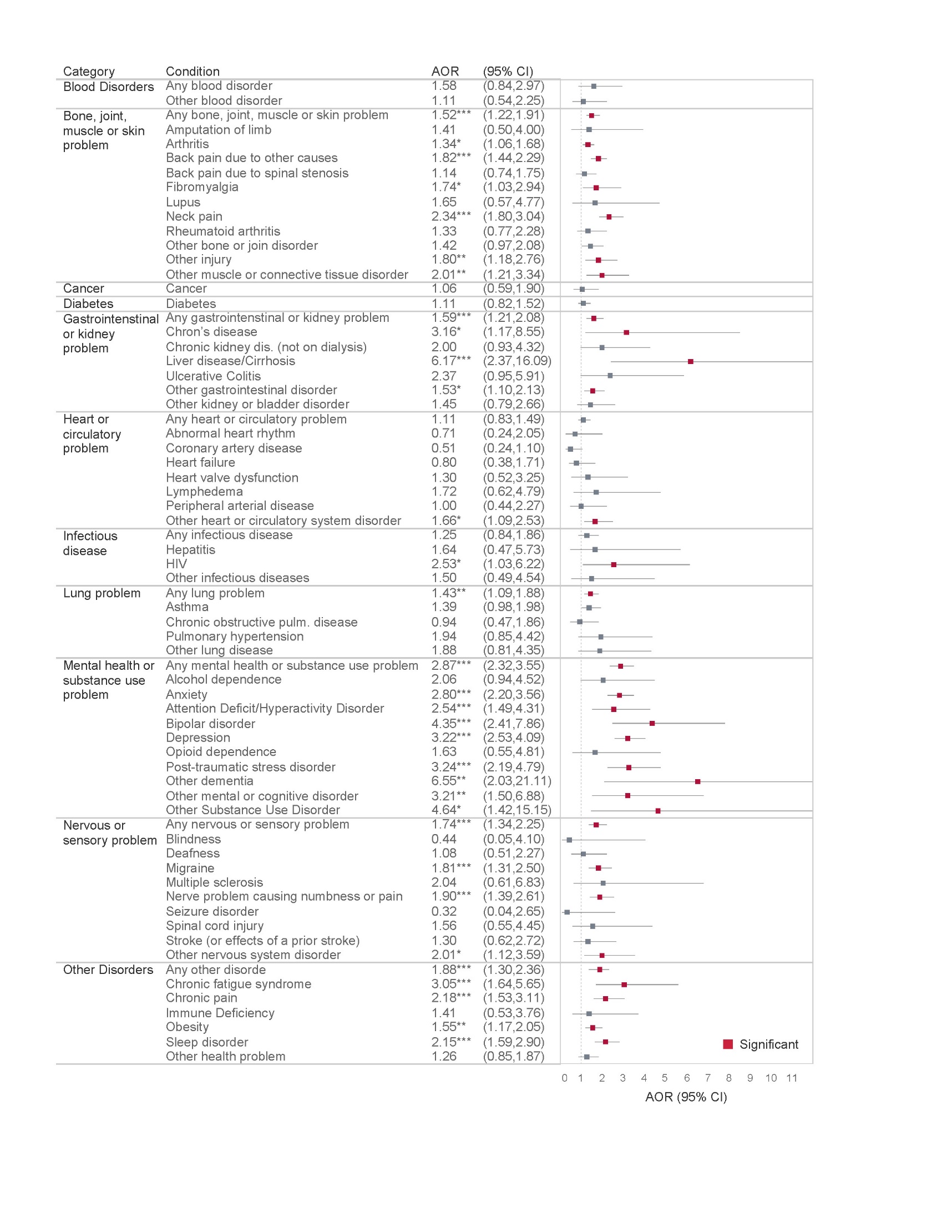

Supplement: Supplementary file 4 [file Data_Sheet_1.DOCX]
